# Postpartum maternal collapse—a first-time presentation of severe mitral stenosis: a case report

**DOI:** 10.1186/s13256-021-02806-5

**Published:** 2021-05-04

**Authors:** Abraham Fessehaye, Yared Teshome Tafere, Desalegn Degafu Abate

**Affiliations:** 1 Department of Obstetrics and Gynecology, Saint Paul’s Hospital millennium Medical College, Addis Ababa, Ethiopia; 2 Tulubolo General Hospital, Tulu Bolo, Ethiopia

**Keywords:** Mitral stenosis, Severe mitral stenosis, Maternal collapse

## Abstract

**Background:**

Among cardiac causes for postpartum maternal collapse, severe mitral stenosis is not listed as a potential cause in current literature. We report a rare case of severe mitral stenosis that presented with severe hypoxia and maternal decompensation in early postpartum period for the first time.

**Case summary:**

A 30-year-old para 2, abortus 1, Ethiopian woman developed severe hypoxia and rapid deterioration on her sixth postoperative day after cesarean delivery for fetal bradycardia with a good fetal outcome. She was put on a mechanical ventilator when she developed respiratory failure. Initially, a diagnosis of pulmonary embolus was considered. After admission to the intensive care unit, severe mitral stenosis was diagnosed with the help of echocardiography. She was managed successfully for congestive heart failure and discharged from the intensive care unit with improvement.

**Conclusion:**

The possibility of mitral stenosis, as a differential diagnosis, should be considered whenever a case of postpartum maternal collapse is encountered. We recommend a routine immediate echocardiography in any patient that experiences postpartum collapse, especially in the presence of a known trigger for heart failure such as long hours of labor, bleeding, anemia, hypotension, and infection.

## Introduction

Postpartum maternal collapse is a rare obstetric condition that carries a high risk of maternal mortality and morbidity. Common causes of maternal collapse include thromboembolic disease, chronic medical conditions, cardiac diseases, and sepsis. Severe mitral stenosis is not listed in the current literature as a potential cause of this critical condition. We report a case of severe mitral stenosis that presented with severe hypoxia and decomposition in the early postpartum period for the first time.

## Case presentation

A 30-year-old gravida 3, para 2, abortus 1 Ethiopian mother was admitted to the labor and delivery ward with a diagnosis of latent first stage of labor and grade-2 meconium-stained amniotic fluid, at cervical dilation of 3 cm and at a gestational age of 40 weeks plus 1 day. She presented with pushing-down pain and passage of liquor of 12 hours duration. She had no history of shortness of breath or history of cough. She had no headache or blurry vision. Her past medical, surgical, family, and psychosocial history was unremarkable. She had no history of prior hospital admission. Upon physical examination at the time of admission, her blood pressure was 140/85 mmHg, pulse rate was 88–92 beats per minute, and respiratory rate was 20 breaths per minute. A diagnosis of preeclampsia was considered, and she was worked up for that. Her complete blood count (CBC) profile was normal with hematocrit of 42%. Her liver enzymes were also normal. Urine protein was negative.

In the second stage of labor, cesarean section was done for an indication of fetal bradycardia, with the outcome being 3200 g alive male neonate with an Apgar score of 7/10 and 8/10 at minutes 1 and 5, respectively. There was postpartum hemorrhage (PPH) due to uterine atony and uterine incision extension, which was managed with a combination of medical management of PPH, compression sutures, and extension repair. She was transfused with two units of blood postoperatively.

Four hours into her postpartum period, she started to experience shortness of breath and her oxygen saturation was 85% on atmospheric oxygen. Her blood pressure was 130/80 mmHg, and her pulse rate was 115 beats per minute. Chest osculation revealed bilateral basal crackles. With a diagnosis of pulmonary edema (caused by preeclampsia), 80 mg of Lasix was given parentally, resulting in a good response. She was taken off oxygen on the following day. Her pulse rate was also in the normal range. Her posttransfusion hematocrit was reported as 24%.

Five days later, she again developed shortness of breath of sudden onset. With severe hypoxia and impending respiratory failure—her oxygen saturation was down to 70% with atmospheric air—pulmonary thromboembolism was considered, and chest computed tomography (CT) scan was considered shortly after she was evaluated by an internal medicine senior resident. She was already on 10 L of 100% oxygen via face mask. Within an hour, while being prepared for imaging and as she was about to be started on empirical anticoagulation therapy, she went into respiratory failure (she could not maintain her saturation with 15 L of 100% oxygen via face mask). Her CBC profile was updated and revealed a hematocrit of 26% and WBC count of 14,400. Her updated liver enzymes and renal function test was otherwise normal.

She was instantly admitted to the intensive care unit (ICU) and put on a mechanical ventilator with the same suspected diagnosis. Meanwhile, a differential diagnosis of heart failure was made, considering the overall course of her medical condition. She was provided 40 mg of Lasix intravenously. She produced adequate urine. Her updated hematocrit was 26.3%. Bedside chest ultrasound and echocardiography was done subsequently. Bilateral pleural effusion was observed with the chest ultrasound, but fluid analysis was unremarkable. The echocardiography findings were severe mitral stenosis (mitral valve area of 0.9 cm^2^) and mild pulmonary hypertension.

With a revised tentative diagnosis of New York Heart Association classification (NYHA) Class IV congestive heart failure secondary to severe mitral stenosis, her Lasix dosage was escalated to 40 mg intravenously three times per day. The patient showed a remarkable improvement. She was extubated after 48 hours of stay in the ICU. She was fully conscious and communicating well when she was transferred to the medical ward for full recovery. Her oxygen saturation was 93% on atmospheric air. The same standing dose of Lasix was continued.

## Discussion

Cardiac disease complicates approximately 1–3% of pregnancies and is responsible for 10–15% of maternal mortality [1, 2]. Cardiac disease in poor countries remains an important cause of maternal mortality. Rheumatic heart disease accounts for most of this mortality, mitral stenosis being the commonest lesion. The overall mortality associated with mitral stenosis is reported to be 1%, increasing to 5% in NYHA classes III and IV [3]. In the presence of atrial fibrillation, maternal mortality is further increased to 14–17% [4]. Concomitant aortic valve disease, anemia, and cardiac arrhythmias may further aggravate the abnormal hemodynamics of mitral stenosis. It is therefore not surprising that mitral stenosis in pregnancy, if not treated appropriately, is associated with high maternal and perinatal mortality [5]. We are reporting a rare presentation of such an alarming cardiac condition, which manifested in the immediate postpartum for the first time.

In the updated literature, differential diagnosis for postpartum collapse includes postpartum hemorrhage, thromboembolic disease, amniotic fluid embolism, drug overdose, intracranial hemorrhage, sepsis, and biochemical causes. Among cardiac causes, acute myocardial infarction, aortic dissection, and cardiomyopathy are the commonly mentioned etiologies. Symptoms and signs indicative of these three cardiac causes include central chest or interscapular pain, a wide pulse pressure, and new cardiac murmur [6].

Contrary to this fact in the current literature, an article dated to 1955 makes the following statement regarding cardiac causes: “It is reported that in the majority of cases where the mother collapses suddenly after childbirth an obstetrical cause is responsible. Nevertheless there is a variety of non-obstetrical causes for such a catastrophe. The main group consists of cases of heart disease, especially mitral stenosis. The woman who is in incipient heart failure before the onset of labor may collapse suddenly when the uterus empties. Here an overloading of the maternal circulation results from the sudden release of blood from the pelvic organs, and this may even be fatal. Other less common cardiovascular diseases include myocarditis, pericarditis, and pericardial effusion” [7].

Retrospectively, the diagnosis of severe mitral stenosis in our case matches with the contents of this statement reported 66 years back, though we could not determine mitral stenosis from the outset. Rather, in our minds, pulmonary embolus was at the top of the list of differentials. This was correct according to common practice and the suddenness of her deterioration, which could not be explained easily by other causes otherwise. Our patient did not have symptoms and signs of heart failure before the index pregnancy and during the index pregnancy, nor even during labor and delivery, and she had no history of prior admission for any cardiac illness. It was her initial good response to the Lasix administration during her first episode of severe hypoxia that brought us to perform an echocardiography immediately, as she went down with the second episode of respiratory distress. The most probable trigger of the heart failure in our case is postpartum hemorrhage requiring blood transfusion.

## Conclusion

While obstetric causes remain the most common, a variety of nonobstetrical causes could lead to postpartum sudden maternal deterioration requiring critical care intervention. From cardiac causes, the possibility of mitral stenosis, as a differential diagnosis, should be considered whenever a case of postpartum maternal collapse is encountered. We recommend a routine immediate echocardiography in any patient that experiences postpartum collapse, especially in the presence of a known trigger for heart failure such as long hours of labor, bleeding, anemia, hypotension, and infection.

## Data Availability

All supporting documents are submitted along with the case report.
